# CircRNA hsa_circ_0074834 promotes the osteogenesis-angiogenesis coupling process in bone mesenchymal stem cells (BMSCs) by acting as a ceRNA for miR-942-5p

**DOI:** 10.1038/s41419-019-2161-5

**Published:** 2019-12-05

**Authors:** Zhengxiao Ouyang, Tingting Tan, Xianghong Zhang, Jia Wan, Yanling Zhou, Guangyao Jiang, Daishui Yang, Xiaoning Guo, Tang Liu

**Affiliations:** 1 Department of Orthopedics, The Second Xiangya Hospital, Central South University, Changsha, Hunan 410011 P.R. China; 2 Department of Immunology, Xiangya School of Medicine, Central South University, 88 Xiangya Rd., Changsha, Hunan 410008 P.R. China

**Keywords:** Regeneration, Trauma

## Abstract

Bone tissue has a strong ability to repair itself. When treated properly, most fractures will heal well. However, some fractures are difficult to heal. When a fracture does not heal, it is called nonunion. Approximately, 5% of all fracture patients have difficulty healing. Because of the continuous movement of the fracture site, bone nonunion is usually accompanied by pain, which greatly reduces the quality of life of patients. Bone marrow mesenchymal stem cells (BMSCs) play an important role in the process of nonunion. Circular RNAs (circRNAs) are a unique kind of noncoding RNA and represent the latest research hotspot in the RNA field. At present, no studies have reported a role of circRNAs in the development of nonunion. After isolation of BMSCs from patients with nonunion, the expression of circRNAs in these cells was detected by using a circRNA microarray. Alkaline phosphatase and Alizarin red staining were used to detect the regulation of osteogenic differentiation of BMSCs by hsa_circ_0074834. The target gene of hsa_circ_0074834 was detected by RNA pull-down and double-luciferase reporter assay. The ability of hsa_circ_0074834 to regulate the osteogenesis of BMSCs in vivo was tested by heterotopic osteogenesis and single cortical bone defect experiments. The results showed that the expression of hsa_circ_0074834 in BMSCs from patients with nonunion was decreased. Hsa_circ_0074834 acts as a ceRNA to regulate the expression of ZEB1 and VEGF through microRNA-942-5p. Hsa_circ_0074834 can promote osteogenic differentiation of BMSCs and the repair of bone defects. These results suggest that circRNAs may be a key target for the treatment of nonunion.

## Introduction

Bone nonunion is a common orthopedic disease. It often occurs in large bone fractures, which limits patient motor ability and brings a heavy financial burden to patients^1,2^. Approximately, 5–10% of fractures will lead to bone nonunion. The primary treatment for nonunion is to improve the biological activity of the bone^1^. Fracture nonunion occurs because the process of fracture healing is affected. At the molecular level, fracture healing includes four main stages: cell recruitment, bone induction, bone regulation and bone conduction. Changes in any process, such as the reduction in bone marrow stromal cells and the low production of bone-inducing matrix, will affect fracture healing and cause nonunion. When a fracture occurs, bone marrow mesenchymal stem cells (BMSCs) migrate to the damaged bone and differentiate into osteoblasts, secreting a large amount of extracellular matrix components to promote fracture healing^3^. The migration and osteogenic differentiation of BMSCs are regulated by various cellular signals^4,5^. BMSCs play an important role in the process of fracture healing. Studies have reported differences in BMSC origin between patients with nonunion and patients with normal bone healing. Hernigou et al.^6^ found that BMSCs in the bone marrow cavity of patients with nonunion decreased in content and distribution, and in vitro studies found that the proliferation of BMSCs from nonunion patients decreased. Seebach et al.^7^ compared the proliferative activity of BMSCs in patients with multiple lesions, single fractures, and atrophic nonunion and found that the proliferation of BMSCs was higher in patients with multiple injuries than in patients with atrophic nonunion. Later, Mathieu et al.^8^ built on previous studies and showed that the proliferative capacity and osteogenic differentiation ability of BMSCs in patients with atrophic nonunion were worse than those of normal BMSCs, but under normal conditions, BMSC function could be restored. At present, the reasons for the decreased proliferation of BMSCs from nonunion patients are still unclear, and further research is needed.

In contrast to traditional linear RNAs (containing 5′ and 3′ ends), circRNA molecules have a closed ring structure, which is not affected by RNA exonuclease, and these molecules are more stably expressed and difficult to degrade. In terms of function, recent studies have shown that circRNA molecules are rich in microRNA binding sites and play the role of microRNA sponges in cells, thereby eliminating the inhibitory effect of the microRNAs on their target genes and thus increasing the expression level of the target genes. This mechanism is called competitive endogenous RNA (ceRNA) mechanism. By interacting with disease-associated microRNAs, circRNA plays an important regulatory role in diseases. CircRNAs have a close relationship with osteogenic differentiation. Zheng et al.^9^ found that the expression of circRNAs in BMSCs changed significantly after differentiation into osteoblasts using a gene chip. Long et al.^10^ found that the expression of circRNAs in mADSCs during osteogenic differentiation was significantly changed by circRNA microarray. In view of these results, we aimed to study the difference in circRNA expression between patients with nonunion and normal fracture healing through microarray. We also explored the role of circRNAs in osteogenic differentiation and bone regeneration of BMSCs.

## Materials and methods

### Isolation and culture of BMSCs

BMSCs were donated by patients who underwent orthopedic surgery after written informed consent. The experiment was approved by the institutional ethics committee of Central South University. Bone marrow aspirate (2 ml) from each donor was inoculated in a 10 cm dish containing medium (DMEM [HyClone, USA], 10% fetal bovine serum [Gibco, USA] and 100 U/ml penicillin G and 100 mg/ml streptomycin [HyClone, USA]) in humidified air at 37 °C. After 3 days of culture, nonadherent cells in the culture supernatant were removed. The BMSCs adhering to the culture dish were cultured in intact medium that was changed every 2 days. When the cultures were confluent, the cells were detached with 0.25% trypsin (Gibco, USA) and then stored or reseeded into culture dishes. The BMSC positive markers including anti-CD90(#555595), anti-CD29(#557332), anti-CD105(#562380), and anti-CD44(#555478), and negative markers, including anti-CD34(#55821) and anti-CD45(#555482), were purchased from BD Biosciences and were used for fluorescence-activated cell sorting (FACS) analysis.

### RNA sequencing

TRIzol solvent was used for isolated total RNA from BMSCs. The RNA was extracted in a 1.5 ml centrifugal tube. After RNA quality inspection, BMSCs were selected and paired paired into 3 pairs. The sequencing was completed by Shanghai Jingneng Biotechnology Co., Ltd., and the sequencing platform was Illumina HiSeq 2500.

### RNA FISH

hBMSCs were cultured on cover slide and washed with CSK buffer after cell growth. hBMSCs were fixed with 4% paraformaldehyde for 10 min at 4 °C, treated with CSK Buffer solution containing 0.5% Triton X-100, 10 mM VRC for 10 min at 4 °C, incubated with 70% alcohol, 4 °C for 10 min. At −20 °C, treated with 70%, 85%, and 100% alcohol for 5 min, respectively. Dehydration and air drying. Then the cell slide was adhered to the slide (face up) with neutral gum. The probe was prepared and the hybridization buffer with the probe was denatured at 76 °C for 10 min. After hybridization, the rubber and cover glass were removed, and the slide was washed with 50% formamide (2 × SSC), 5 min × 3, and then 2 × SSC was washed at 42 °C, 5 min × 3. DAPI was re-dyed with 5 min × 3. DAPI. The appropriate amount of DAPI storage solution was obtained by phosphate-buffered saline (PBS). Diluted to 1 µg/ml of working fluid, inhale 5 µl droplets on the slide, cover the slide, incubate at dark room temperature for 5 min. Remove the cover glass and wash it twice in PBS. Remove excessive liquids and add antifade reagent seal. Observation under fluorescence microscope.

### Lentivirus-mediated circRNA has_circ_0074834 overexpression and knockdown

The circRNA hsa_circ_0074834 (Position chr5:159437506-159492550; Genomic length: 55044;Spliced sequence length:1386 bp; Best transcript: NM_003314; Gene symbol: TTC1.) was ligated into the pCDH-cirRNA vector using PCR primers to amplify the cDNA region. Linear hsa_circ_0074834 with 12× MS2b tig.The The circRNA hsa_circ_0074834 shRNA was ligated into the pLKO.1 vector using PCR primers to amplify the shRNA. The pCDH-cirRNA and pCDH-cirRNA-hsa_circ_0074834 or pLKO.1-Vector and pLKO.1-hsa_circ_0074834 constructs were transfected into the HEK293T viral packaging cell line together with pSPAX2 and pMD2G. Viral supernatant was used for the infection of BMSCs.

### qRT-PCR analysis

Total RNA was extracted using TRIzol reagent, cDNA was obtained by reverse transcription using the PrimeScript RT Master Mix cDNA Synthesis Kit, and quantitative real-time polymerase chain reaction (qRT-PCR) was performed using the SYBR Green PCR kit. The qRT-PCR reaction conditions were set as follows: denaturation at 95 °C for 30 s, 50 cycles at 95 °C for 10 s, and 60 °C for 30 s. All measurements were calculated using the 2^−ΔΔCT^ method with GAPDH as an endogenous control. qRT-PCR was performed with 0.2 μl cDNA samples with the following primers: RUNX2: forward, 5′-TACCTGAGCCAGATGACG-3′; reverse, 5′-CAGTGAGGGATGAAATGC-3′; COL1A1: forward, 5′-GCCGTGACCTCAAGATGTG-3′; reverse, 5′-GCCGAACCAGACATGCCTC-3′; OCN: forward, 5′-TGAGAGCCCTCACACTCCTC-3′; reverse, 5′-ACCTTTGCTGGACTCTGCAC-3′; GAPDH: forward, 5′-GTTCCAATATGATTCCACCC-3′; reverse, 5′-AGGGATGATGTTCTGGAGAG-3′.

### RNA immunoprecipitation (RIP)

The cells were harvested by trypsinization and resuspended in PBS-based freshly prepared nuclear isolation buffer (2 mL) and water (6 mL). The cells were kept on ice for 20 min (with frequent mixing). The nuclei were pelleted by centrifugation at 2500 × *g* for 15 min. The nuclear pellet was resuspended in freshly prepared RIP buffer (1 mL). The resuspended nuclei were split into two fractions of 500 mL each (for mock and IP). Chromatin was mechanically sheared using a Dounce homogenizer with 15–20 strokes. The nuclear membrane and debris were pelleted by centrifugation at 13,000 rpm for 10 min. Antibody to MS2b (10 µg) was added to the supernatant (10 mg) and incubated for 2 h (to overnight) at 4 °C with gentle rotation. Protein A/G beads (40 µL) were added to the mixture and incubated for 1 h at 4 °C with gentle rotation. Beads were pelleted at 2500 rpm for 30 s, the supernatant was removed, and the beads were resuspended in 500 mL RIP buffer. This process was repeated for a total of three RIP washes, followed by one wash in PBS. Coprecipitated RNAs were isolated by resuspending the beads in TRIzol RNA extraction reagent.

### Western blot analysis

Total protein was extracted by RIPA, and protein concentration was detected by a bicinchoninic acid protein quantification kit^11,12^. A 30 μg protein sample was used for 10% sodium dodecyl sulfate polyacrylamide gel electrophoresis. After electrophoresis, the protein was transferred to a polyvinylidene fluoride (PVDF) membrane, and the PVDF membrane was blocked with 5% bovine serum albumin. Then, primary antibody was added and incubated overnight, after which an incubation with an HRP-labeled secondary antibody and ECL development were performed. The following primary antibodies were used in this study: COL1A1 (Abcam, #ab34710), RUNX2 (Abcam, #ab192256), OCN (Abcam, #ab13418) ZEB1 (Abcam, #ab245283), VEGF (Abcam, #ab52917), beta-catenin (Abcam, #ab32572), Dicer (Abcam, #ab227518), and GAPDH (Abcam, #ab181602).

### Osteogenic differentiation assay

The cells were washed twice with PBS, fixed with 4% paraformaldehyde for 15 min, and then stained with ALP staining solution or Alizarin red staining solution for 30 min at 37 °C^13^. After staining, the cells were washed twice with PBS and photographed. Quantitative analysis of ALP activity, digestion of the cells by trypsin, and collection of the cells were performed according to the manufacturer instruction for the ALP activity quantification kit. Absorbance was measured at 450 nm. Semiquantitative analysis of Alizarin red staining was performed by the addition of 1 ml of 0.1 N NaOH and detection of absorbance at 480 nm.

### HUVEC scratch test

The cells were seeded at a density of 1 × 10^5^ cells/well into a 12-well culture plate and cultured for 12 h using serum-free medium. After a pipette tip scratch, the suspension cells were washed away with medium, and the remaining cells were photographed at 0 and 24 h.

### HUVEC Transwell migration assay

A Transwell migration assay was performed using Transwell inserts (BD Biosciences, USA) with an 8 μm pore filter. First, 5 × 10^4^ cells in serum-free medium were seeded into the upper chamber of the insert precoated with Matrigel, and 700 µl conditional medium was added to the lower chamber. After 24 h of incubation, the cells were fixed with 75% ethanol and stained with crystal violet. Then, cells on the top surface of the membrane were carefully wiped off, and cells on the lower surface were examined with a microscope. Five random fields were photographed for counting purposes, and the average number of migrated cells was used as a measure of migration capacity.

### HUVEC tube formation assay

HUVECs were serum-starved for 16 h and then seeded at a density of 4 × 10^4^ per well on growth factor-depleted Matrigel (BD Biosciences, NSW, Australia) in 48-well plates. Conditional medium was added, and the results were quantified 4 h later. Microscopic fields containing the tube structures formed in the gel were photographed at low magnification (×10). Five fields per test condition were examined.

### Ectopic bone formation analysis

Subcutaneous stem cell implantation was performed as previously described^14,15^. Briefly, the BMSCs were infected with different lentiviruses. Twenty-four hours after infection, cells were collected, and approximately 3.0 × 10^6^ cells were mixed with β-TCP ceramic particles (50 mg, Shanghai Bio-lu Biomaterials Co., Ltd., Shanghai, China). This mixture was subcutaneously implanted into the dorsal surface of nude mice. After 6 or 12 weeks, the implants were harvested and fixed in 4% paraformaldehyde, decalcified with an ultrasonic decalcification instrument, and then embedded in paraffin. For histological analyses, the sections (5 μm) were stained with hematoxylin and eosin (H&E).

### Femoral monocortical defect model

The femoral monocortical defect model was performed as previously described^16,17^. Briefly, nude mice were placed under general anesthesia, and then, the lateral aspect of the right femur was exposed and the overlying soft tissues were pushed aside while carefully preserving the periosteum. A monocortical osseous hole (0.8 mm diameter) was created on the femur lateral surface using a round burr attached to a dental drill. Irrigation with saline was used to remove bone dust and fragments. Approximately, 5.0 × 10^4^ hBMSCs, which were obtained after different treatments for 24 h, were resuspended in a mixture of medium and Matrigel and then transplanted into the osseous hole. One month later, right femurs were fixed in 4% paraformaldehyde for 24 h at 4 °C. The samples were scanned by high-resolution microcomputed tomography (µCT) (SCANCO, Switzerland) with a spatial resolution of 5 μm. Sagittal image sections of model femurs were used to perform 3D histomorphometric analysis. We defined the regions of interest as (i) the hole region between the interrupted cortical bone ends, (ii) injured bone marrow, and (iii) periosteal callus outside the hole. Old bone fragments remaining from the drilling were excluded from the regions of interest. A total of 100 consecutive images were used for 3D reconstruction and analysis, covering most of the injured region and periosteal callus. Structural parameters measured included BMD and bone volume percentage (BV/TV).

### Statistical analysis

Statistical significance was assessed using two-tailed Student’s *t* test or analysis of variance^18^. All statistical analyses were performed using SPSS software version 19.0^19^. Statistical significance was considered at *p < 0.05 and **p < 0.01. Data are presented as the mean ± S.D.

## Results

### CircRNA hsa_circ_0074834 expression is downregulated in BMSCs isolated from bone nonunion patients

**Fig. 1:**
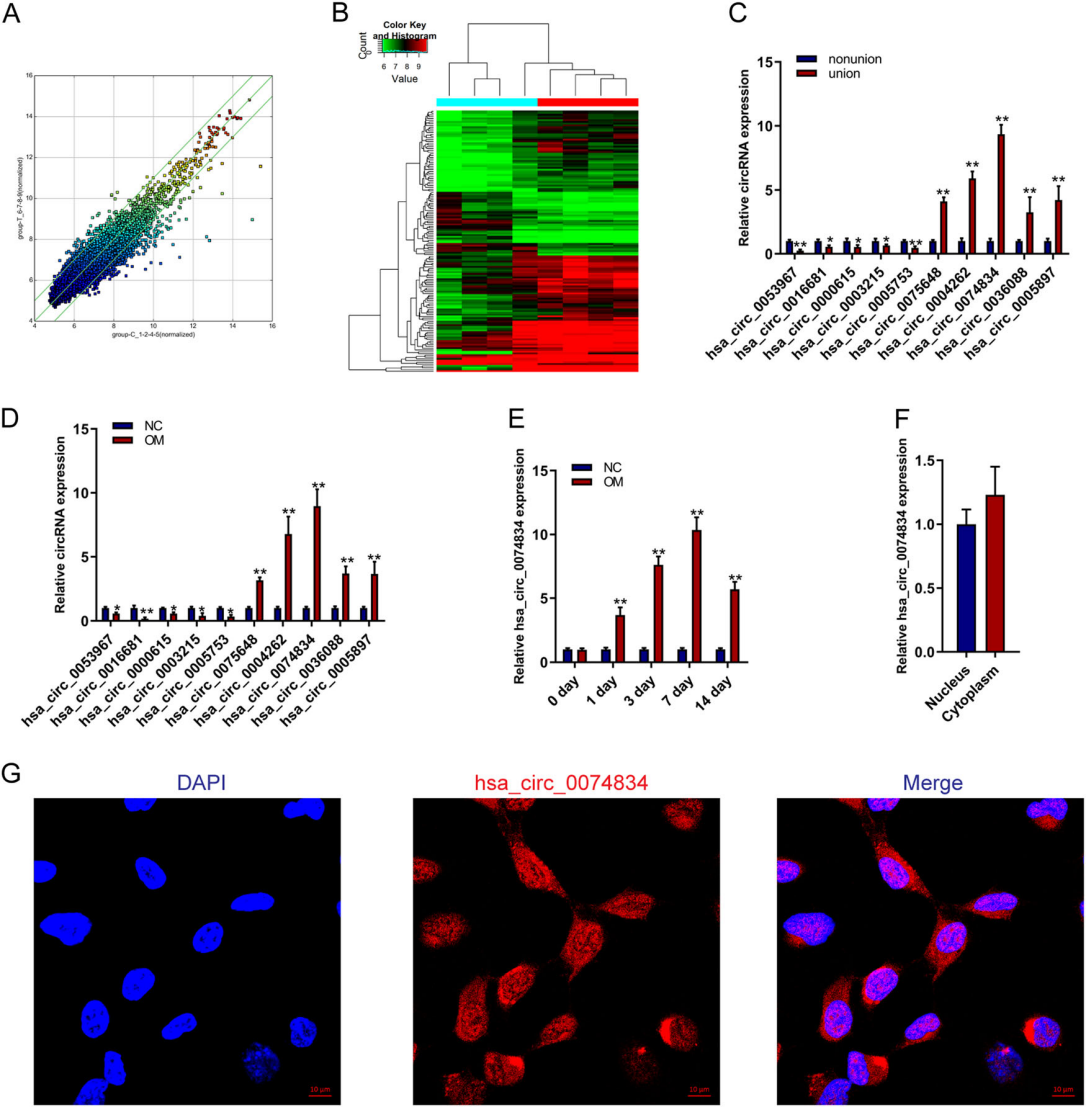
CircRNA hsa_circ_0074834 is downregulated in BMSCs isolated from bone nonunion patients. **a** Scatter map of circRNA expression in BMSCs isolated from patients with nonunion or normal bone fracture healing. **b** Heatmap of circRNA expression in BMSCs isolated from patients with nonunion or normal bone fracture healing. **c** qRT-PCR analysis of circRNA expression in BMSCs isolated from patients with nonunion or normal bone fracture healing. **d** qRT-PCR analysis of circRNA expression in BMSCs on the third day of osteogenesis. **e** qRT-PCR analysis of circRNA hsa_circ_0074834 expression in BMSCs during osteogenesis at 0, 1, 3, 7, and 14 days. **f** qRT-PCR analysis of circRNA hsa_circ_0074834 expression in the nucleus and cytoplasm of BMSCs. **g** Fluorescence in situ hybridization (FISH) analysis of circRNA hsa_circ_0074834 in BMSCs. Scale bar 10 μm. The results are presented as the mean ± SD. *p < 0.05, **p < 0.01.

To study the mechanism of circRNA in the occurrence of bone nonunion, we isolated BMSCs from patients with normal fracture healing and nonunion. The expression of BMSCs surface markers was also detected by FACS (Fig. S1). The expression of circRNAs in the BMSCs were analyzed by microarray. The results showed that the expression of circRNAs was largely different in BMSCs from patients with nonunion and normal fracture healing (Fig. 1a, b). The results of the microarray analysis were verified by qRT-PCR and showed that the expression of hsa_circRNA_0074834 was significantly increased in BMSCs isolated from normal bone fracture healing patients compared with bone nonunion patients (Fig. 1c). BMSCs were isolated and cultured for osteogenic differentiation, and the expression of circRNAs was detected on the third day of osteogenic differentiation. The results showed that hsa_circRNA_0074834 expression increased most significantly during BMSC osteogenic differentiation (Fig. 1d). Further, we examined the expression changes of hsa_circ_0074834 in the osteogenic differentiation of BMSCs, and the results showed a significant increase during osteogenic differentiation (Fig. 1e). To further explore the function of hsa_circRNA_0074834, we found that it was evenly distributed in the nucleus and cytoplasm using qRT-PCR and fluorescence in situ hybridization (Fig. 1f, g).

### CircRNA hsa_circ_0074834 promotes osteogenesis of BMSCs

**Fig. 2:**
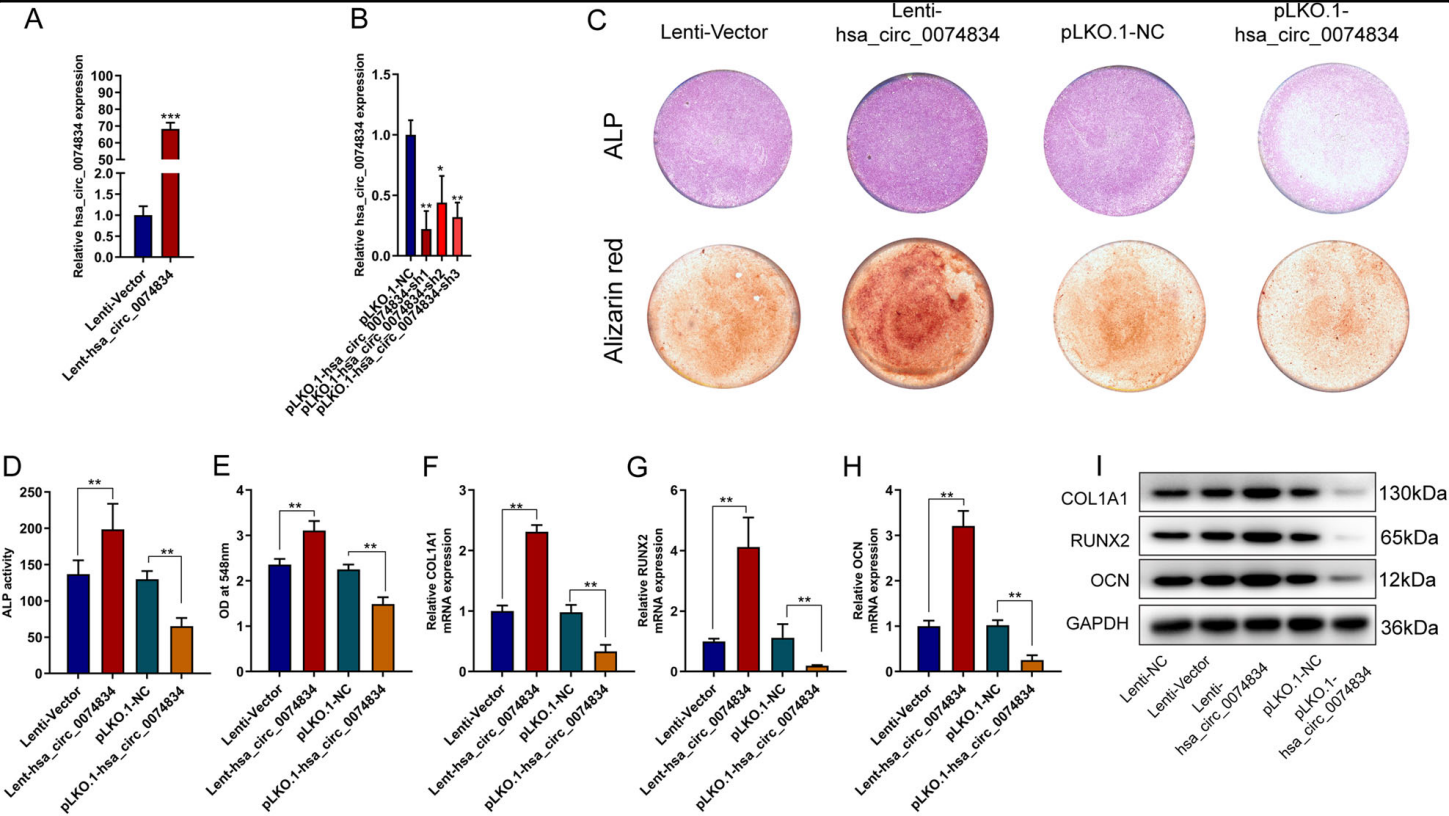
CircRNA hsa_circ_0074834 promotes BMSC osteogenesis. qRT-PCR analysis of the circRNA hsa_circ_0074834 expression in BMSCs after transfection with lentiviruses Lenti-hsa_circ_0074834 (**a**) and pLKO.1-hsa_circ_0074834-sh1, pLKO.1- hsa_circ_0074834-sh2 and pLKO.1-hsa_circ_0074834-sh3 (**b**). **c** ALP and Alizarin red staining after BMSCs were transfected with lentiviruses Lenti-hsa_circ_0074834 and pLKO.1-hsa_circ_0074834 on the 7th day and 14th day, respectively. Quantitative analysis of (**d**) ALP and (**e**) Alizarin red staining after BMSCs were transfected with lentiviruses Lenti-hsa_circ_0074834 and pLKO.1-hsa_circ_0074834 on the 7th and 14th days, respectively. qRT-PCR analysis of COL1A1 (**f**), RUNX2 (**g**), and OCN (**h**) mRNA expression in BMSCs after transfection with lentiviruses Lenti-hsa_circ_0074834 and pLKO.1-hsa_circ_0074834. **i** Western blot analysis of COL1A1, RUNX2, and OCN expression after transfection with lentiviruses Lenti-hsa_circ_0074834 and pLKO.1-hsa_circ_0074834. The results are presented as the mean ± SD. *p < 0.05, **p < 0.01.

We overexpressed and knocked down circRNA hsa_circRNA_0074834 in BMSCs by lentivirus. qRT-PCR results showed that the overexpression lentivirus significantly increased the expression of hsa_circRNA_0074834 in BMSCs (Fig. 2a), and the knockdown lentivirus significantly decreased the expression of hsa_circRNA_0074834 in BMSCs (Fig. 2b). Overexpression of hsa_circRNA_0074834 enhanced ALP staining and ALP activity, and its knockdown inhibited ALP staining and ALP activity (Fig. 2c, d). Increased expression of hsa_circRNA_0074834 promoted the formation of calcium nodules in BMSCs, and decreased expression of hsa_circRNA_0074834 inhibited the formation of calcium nodules (Fig. 2c, e). Hsa_circRNA_0074834 overexpression induced the expression of COL1A1, RUNX2 and OCN at the mRNA and protein levels, and the opposite was observed with circRNA knockdown (Fig. 2f–i). The gene symbol of hsa_circRNA_0074834 was TTC1. Two linear transcripts of TTC1 gene were NM_001282500.1 and NM_003314.3, respectively. We overexpressed NM_001282500.1 and NM_003314.3 in BMSCs, respectively. The results showed that the overexpression of NM_001282500.1 and NM_003314.3 had no effect on the osteogenic differentiation of BMSCs (Fig. S2A–D), and the expression of TTC1 did not change during the osteogenic differentiation of BMSCs detected by Western blot (Fig. S2E). CCK-8 assay showed that hsa_circ_0074834 had no effect on the proliferation of hBMSCs (Fig. S3).

### CircRNA hsa_circ_0074834 functions as a ceRNA for miR-942-5p to regulate the expression of ZEB1 and VEGF

**Fig. 3:**
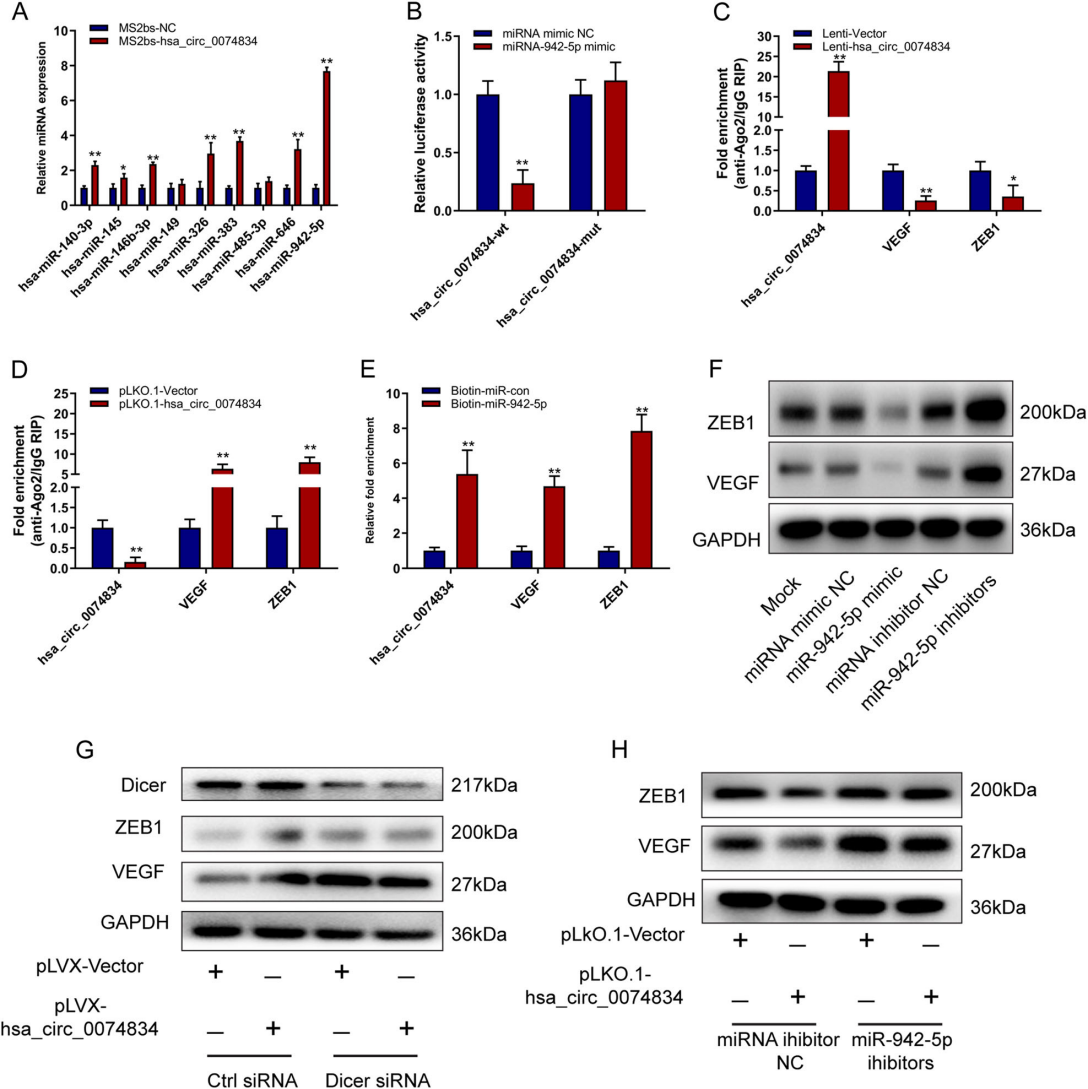
CircRNA hsa_circ_0074834 functions as a ceRNA for miR-942-5p to regulate the expression of ZEB1 and VEGF. **a** MS2bs-based RIP assay in BMSCs 48 h after transfection with MS2bp-YFP plasmid along with MS2bs-linear-hsa_circ_0074834 or MS2bs-Rluc (control vectors). **b** Luciferase activity of psiCHECK2-hsa_circ_0074834-wt and psiCHECK2-hsa_circ_0074834-mut upon cotransfection with miRNA mimics NC or miR-942-5p mimics in 293T cells. **c** RIP assay of the enrichment of Ago2 with hsa_circ_0074834, ZEB1, and VEGF transcripts relative to IgG in BMSCs transfected with Lenti-Vector or Lenti-hsa_circ_0074834. **d** RIP assay of the enrichment of Ago2 with hsa_circ_0074834, ZEB1, and VEGF transcripts relative to IgG in BMSCs transfected with pLKO.1-Vector or pLKO.1-hsa_circ_0074834. **e** miRNA-RIP assay of the enrichment of hsa_circ_0074834, ZEB1 and VEGF transcript on miR-942-5p relative to miRNA mimic NC in BMSCs transfected with Biotin-miR-con or Biotin-miR-942-5p. **f** Western blot analysis of ZEB1 and VEGF expression in BMSCs transfected with miR-942-5p mimics, miR-942-5p inhibitors or miRNA mimics NC and miRNA inhibitors NC. **g** Western blot analysis of ZEB1 and VEGF expression in BMSCs cotransfected with lentivirus Lenti-Vector or Lenti-hsa_circ_0074834 along with Dicer ctrl siRNA or Dicer siRNA. **h** Western blot analysis of ZEB1 and VEGF expression in BMSCs cotransfected with Lenti-Vector or Lenti-hsa_circ_0074834 along with Dicer ctrl siRNA or Dicer siRNA. **h** Western blot analysis of ZEB1 and VEGF expression in BMSCs cotransfected with pLKO.1-Vector or pLKO.1-hsa_circ_0074834 along with miRNA inhibitors NC or miR-942-5p inhibitors. The results are presented as the mean ± SD. *p < 0.05, **p < 0.01.

To further investigate the mechanism by which circRNA hsa_circ_0074834 regulates the osteogenic differentiation of BMSCs, we used bioinformatics methods to predict the miRNAs that bind to hsa_circ_0074834. Using an MS2bs-based RIP assay, we found that miR-942-5p could bind to the circRNA (Fig. 3a and Fig. S4A). We mutated the binding site and constructed a luciferase reporter plasmid. The luciferase assay also showed that miR-942-5p could combine with circRNA hsa_circ_0074834 (Fig. 3b). Using bioinformatics, miR-942-5p was found to target the ZEB1 and VEGF 3′-UTR regions (Fig. S4B) and the luciferase assay also showed that miR-942-5p could combine with ZEB1 and VEGF 3′-UTR regions (Fig. S4C, D).Using bioinformatics, miR-942-5p was found to target the ZEB1 and VEGF 3′-UTR regions. We used the Ago2 RIP assay to find that ZEB1 and VEGF compete with circRNA hsa_circ_0074834 for binding to Ago2 (Fig. 3c, d). On the other hand, we found that miR-942-5p can be combined with ZEB1, VEGF, and circRNA hsa_circ_0074834 by Biotin-miRNA RIP assay (Fig. 3e). miR-942-5p can significantly reduce the expression of ZEB1 and VEGF protein levels, and inhibition of miR-942-5p function can promote the expression of ZEB1 and VEGF (Fig. 3f). After the suppressed expression of Dicer, the effect of circRNA hsa_circ_0074834 on the expression of ZEB1 and VEGF was inhibited and suppressed the function of miR-942-5p. The effect of circRNA hsa_circ_0074834 on ZEB1 and VEGF expression was also reduced.

### CircRNA hsa_circ_0074834 promotes BMSC osteogenesis through ZEB1

**Fig. 4:**
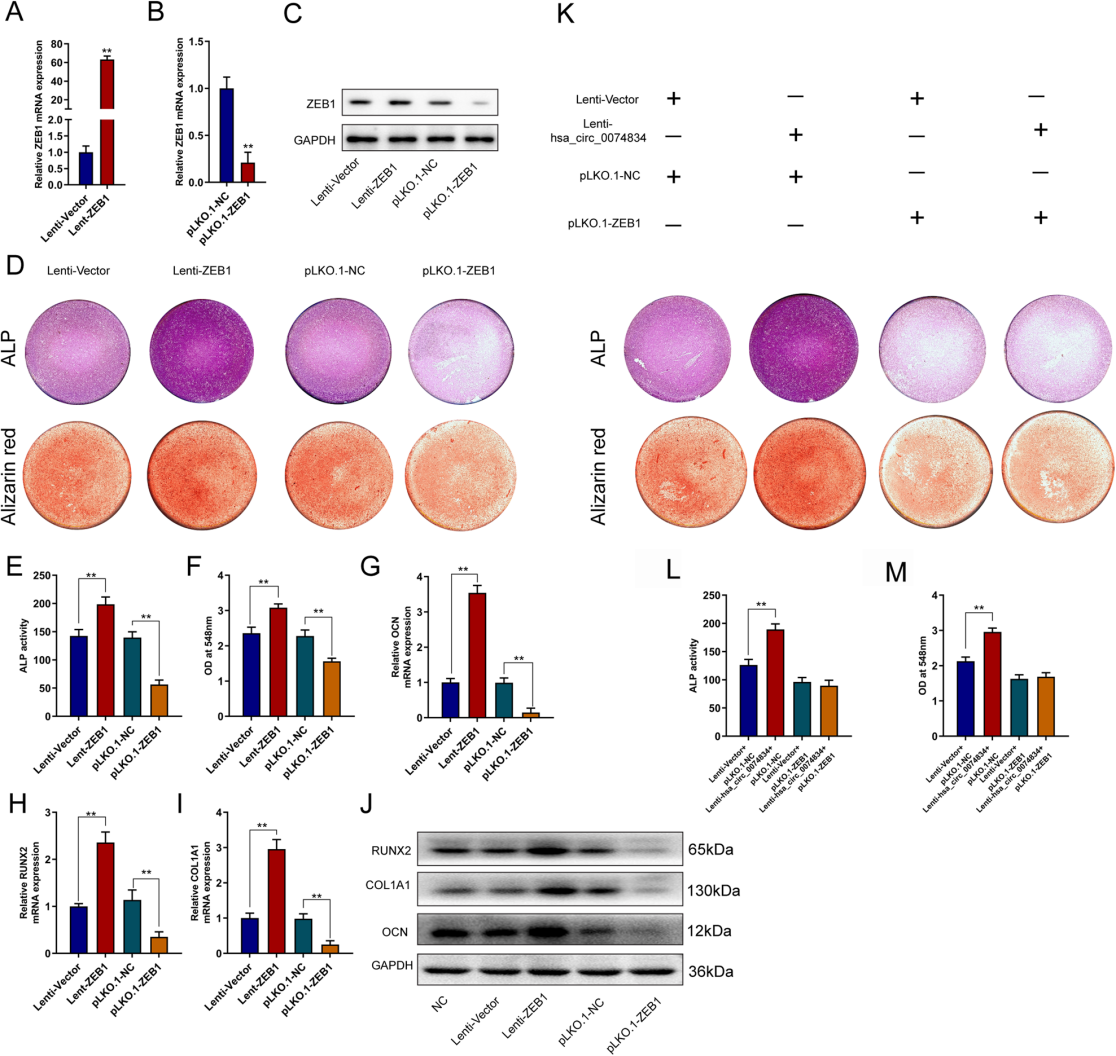
CircRNA hsa_circ_0074834 promotes BMSC osteogenesis through ZEB1. qRT-PCR analysis of ZEB1 mRNA expression in BMSCs after transfection with lentiviruses Lenti-ZEB1 (**a**) and pLKO.1-ZEB1 (**b**). **c** Western blot analysis of ZEB1 protein expression after transfection with lentiviruses Lenti-ZEB1 and pLKO.1-ZEB1. **d** ALP and Alizarin red staining after BMSCs were transfected with lentiviruses Lenti-ZEB1 and pLKO.1-ZEB1 on the 7th day and 14th day. Quantitative analysis of (**e**) ALP and (**f**) Alizarin red staining after BMSCs were transfected with lentiviruses Lenti-ZEB1 and pLKO.1-ZEB1 on the 7th day and 14th day. qRT-PCR analysis of OCN (**g**), RUNX2 (**h**), and COL1A1 (**i**) mRNA expression in BMSCs after transfection with lentiviruses Lenti-ZEB1 and pLKO.1-ZEB1. **j** Western blot analysis of RUNX2, COL1A1, and OCN protein expression after transfection with lentiviruses Lenti-ZEB1 and pLKO.1-ZEB1. **k** ALP and Alizarin red staining after BMSCs were cotransfected with lentiviruses Lenti-Vector and Lenti-hsa_circ_0074834 along with lentivirus pLKO.1-Vector or pLKO.1-ZEB1 on the 7th day and 14th day, respectively. Quantitative analysis of (**l**) ALP and (**m**) Alizarin red staining after BMSCs were cotransfected with lentivirus Lenti-Vector and Lenti-hsa_circ_0074834 along with lentivirus pLKO.1-Vector or pLKO.1-ZEB1 on the 7th day and 14th day, respectively. The results are presented as the mean ± SD. *p < 0.05, **p < 0.01.

To detect the function of ZEB1 in the osteogenic differentiation of BMSCs, we overexpressed and knocked down ZEB1 in BMSCs by lentivirus. The qRT-PCR results showed that the ZEB1 overexpression lentivirus could significantly increase the expression of ZEB1 in BMSCs (Fig. 4a, c), and the ZEB1 knockdown lentivirus could significantly decrease the expression of ZEB1 in BMSCs (Fig. 4b, c). Overexpression of ZEB1 enhanced ALP staining and ALP activity, and knockdown of ZEB1 inhibited ALP staining and ALP activity (Fig. 4d, e). Increased expression of ZEB1 promoted the formation of calcium nodules in BMSCs, and decreased expression of ZEB1 inhibited the formation of calcium nodules (Fig. 4d, f). ZEB1 overexpression promoted the expression of COL1A1, RUNX2 and OCN at the mRNA and protein levels and vice versa (Fig. 2g–j). When the expression of ZEB1 was inhibited, the enhancement effect of circRNA hsa_circ_0074834 on ALP activity and calcium nodule content during BMSC osteogenic differentiation was suppressed (Fig. 4k–o).

### CircRNA hsa_circ_0074834 promotes the osteogenesis-angiogenesis coupling process

**Fig. 5:**
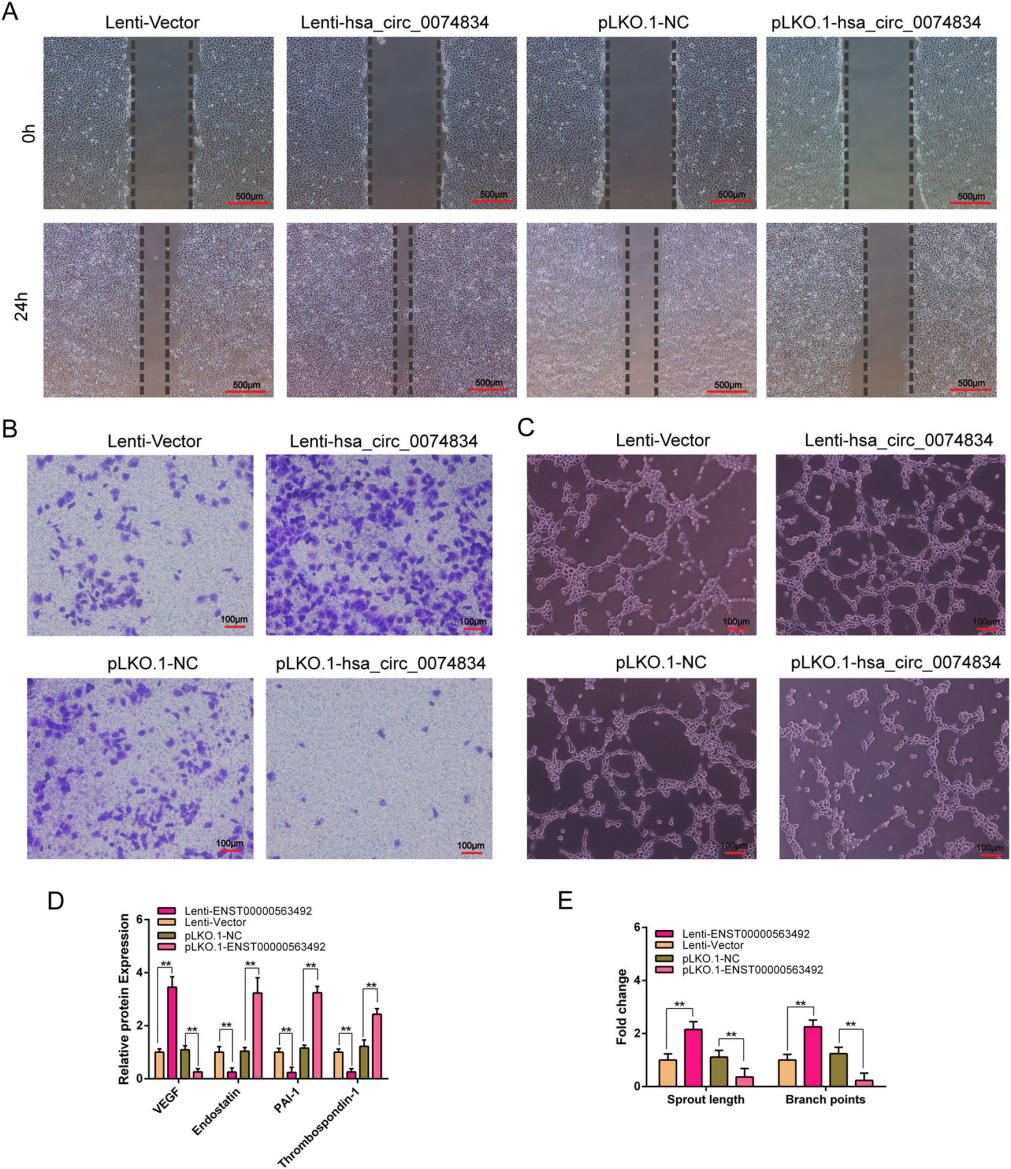
CircRNA hsa_circ_0074834 promotes angiogenesis of HUVECs. **a** The scratch test was used to detect the migration ability of HUVECs, and photographs were taken at 0 and 24 h after adding the conditioned medium from BMSCs transfected with lentivirus Lenti-hsa_circ_0074834 or pLKO.1-hsa_circ_0074834 and control plasmid. Scale bar 500 μm. **b** Transwell test was used to detect the invasion ability of HUVECs, and photographs were taken at 24 h after adding the conditioned medium from BMSCs transfected with lentivirus Lenti-hsa_circ_0074834 or pLKO.1-hsa_circ_0074834 and the control plasmid in the down chamber. Scale bar 100 μm. **c** Tube formation analysis of angiogenesis of HUVECs and **e** quantitative analysis. Scale bar 100 μm. **d** ELISA analysis of the secretion of VEGF, Endostatin, PAI-1 and Thrombospondin-1 in the culture medium of BMSCs transfected with lentivirus Lenti-hsa_circ_0074834 or pLKO.1-hsa_circ_0074834 and control plasmid. The results are presented as the mean ± SD. *p < 0.05, **p < 0.01.

Overexpression and knockdown of circRNA hsa_circ_0074834 in BMSCs followed by osteogenic differentiation, HUVECs were cultured using induced differentiation culture supernatants for scratch, Transwell and tube formation experiments. Scratch experiments showed that overexpression of circRNA hsa_circ_0074834 in the culture supernatant enhanced HUVEC migration, whereas inhibition of circRNA hsa_circ_0074834 expression in the culture supernatant reduced HUVEC migration (Fig. 5a). Transwell experiments showed that overexpression of hsa_circ_0074834 in the culture supernatant enhanced HUVEC invasion, and conversely, inhibition of hsa_circ_0074834 expression in the culture supernatant reduced the invasion of HUVECs (Fig. 5b). Angiogenesis experiments showed that overexpression of hsa_circ_0074834 in the culture supernatant enhanced the angiogenesis of HUVECs, and conversely, inhibition of the expression of hsa_circ_0074834 suppressed the angiogenesis of HUVEC (Fig. 5c, d). Based on ELISA measurements of proangiogenic and angiogenic factor expression in the culture supernatant, overexpression of hsa_circ_0074834 increased the expression of VEGF, in contrast to inhibition of hsa_circ_0074834 expression that reduced the expression of VEGF (Fig. 5e).

### CircRNA hsa_circ_0074834 improved BMSC osteogenesis and bone regeneration in vivo

**Fig. 6:**
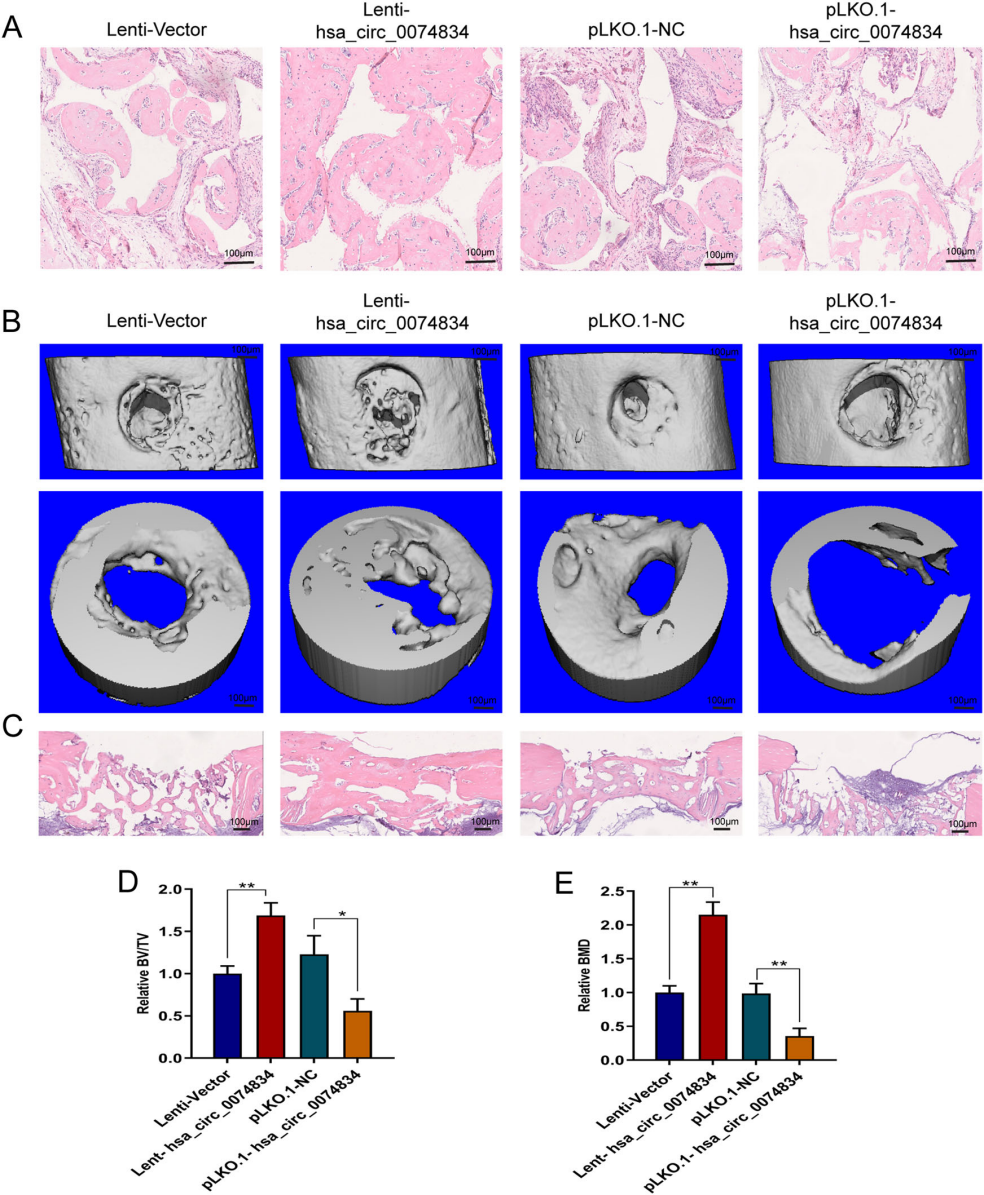
CircRNA hsa_circ_0074834 improves BMSC osteogenesis and bone regeneration in vivo. **a** Representative images of H&E staining show new bone formation in ectopic osteogenesis (magnification: ×100). Scale bar 100 μm. **b** Lateral views of a 3D reconstruction of injured femoral (top panel) and mineralized bone formed in the hole region (lower panel) by μCT. Scale bar 100 μm. **c** Representative images of H&E staining show new bone formation in the bone defect position (magnification: ×40). Scale bar 100 μm. **d** Relative 3D reconstruction parameters BV/TV of mineralized bone formed in the hole region by μCT. **e** Relative 3D reconstruction parameters BMD of mineralized bone formed in the hole region by μCT. The results are presented as the mean ± SD. *p < 0.05, **p < 0.01.

To detect the effect of circRNA hsa_circ_0074834 on bone regeneration, we used an ectopic osteogenesis model to demonstrate that hsa_circ_0074834 overexpression can significantly promote the osteogenic differentiation of BMSCs in vivo, and a decrease in the expression of hsa_circ_0074834 can inhibit the osteogenic differentiation of BMSCs in vivo (Fig. 6a). In addition, it was found that hsa_circ_0074834 overexpression can significantly promote bone regeneration and that decreased expression of hsa_circ_0074834 can inhibit bone regeneration in a bone defect model (Fig. 6b–e).

## Discussion

Nonunion is a common late complication in patients with clinical fractures. It generally refers to a fracture that has not healed 6–9 months after the occurrence and in which there has been no sign of healing after 3 months of continuous observation. Nonunion is observed when the fracture crevice and callus remain unchanged, accompanied by a clear fracture line, separation, no bridging bone trabecula, visible bone end sclerosis, bone marrow cavity closure or fracture end atrophy, and the fracture end usually has abnormal activity and limb pain. After fracture, the factors affecting fracture healing, include the incision site of the fracture, the presence of severe open injury, infection, diabetes, malnutrition and cachexia, and tobacco and drug use^20^. In addition, researchers have gradually improved their understanding of the metabolism of new bone tissue and believe that it will be an important part of fracture treatment. Studies have shown that serum 25-hydroxyvitamin D deficiency (<30 ng/mL) or serum 25-hydroxyvitamin D deficiency (<20 ng/mL) are risk factors for nonunion. Various studies have shown that smoking increases the risk of nonunion, blocks small blood vessels and increases the incidence of infection^21,22^.

At present, research studies have mainly focused on the treatment of bone nonunion by BMSCs, but there are relatively few reports on the function of BMSCs in the process of bone nonunion. Zhang et al. found that the osteogenic differentiation ability of BMSCs from patients with bone nonunion is weakened, but the specific mechanism is less studied^23^. For the first time, we found that circRNA hsa_circ_0074834 was downregulated in BMSCs from patients with nonunion by high-throughput assay and could regulate the osteogenic differentiation of BMSCs. It was also the first time that a circRNA related to nonunion was found. To date, four main biological functions of circRNAs have been characterized. CircRNAs have been shown to participate in the regulation of transcription in the nucleus. It was found that knockout of circRNAs resulted in a significant decrease in the expression of their parent genes, and circRNAs could cis-regulate the expression of their target genes by positively regulating the transcription of RNA polymerase II. In competition with precursors for transcription, circRNAs originate from RNA precursors and can be produced by typical linear splicing or atypical splicing during processing. Increasing the efficiency of typical linear splicing will result in a significant reduction in the number of circRNAs. In longer introns the efficiency of typical linear splicing decreases significantly, while the efficiency of cyclization increases significantly. This result indicates that circRNAs can compete with the RNA precursor during transcription. Targeted binding sites of miRNAs competing with RNA in the cytoplasm, Except for a small number of circRNAs produced by intron cyclization in the nucleus, most of the circRNAs are mainly located in the cytoplasm. In recent years, a large number of studies have found that circRNAs located in the cytoplasm can compete with RNAs for the target binding sites of miRNAs, thus regulating the expression of the mRNAs. One study found that circRNA ciRS-7 is produced from the sense strand of CDR1as, which contains more than sixty conserved miRNA miR-7 target binding sites and is highly abundantly expressed in the cytoplasm. CiRS-7 can adsorb more than 20,000 miR-7 molecules in each cell, so it can compete with mRNA for the target binding site of miR-7, thus affecting gene expression^24^. The interaction between circRNAs and miRNAs has become a hot topic in recent years, but only a small number of circRNAs have been identified to contain multiple targeted binding sites for miRNAs. Contains a ribosome entry site that can be translated to express an effective protein. CircRNAs were originally defined as noncoding RNAs. With the rapid development of bioinformatics and high-throughput sequencing technologies, it has been found that circ-ZNF609, which is produced by the cyclization of exon 2 of the ZNF609 gene, contains an initiation codon and a stop codon in the open reading frame. Further proteomics analysis and Western blot verification revealed that endogenous circ-ZNF609 has protein coding potential, but the translation efficiency is two orders of magnitude lower than that for linear RNA^25^. In addition, studies have reported that in the brain tissue of Drosophila, some circRNAs can bind to ribosomes by ribosome imprinting. For example, the stop codon of circMbl contains a ribosome binding site, which allows the circRNA to be translated and express proteins^26^. The discovery of the ability of circRNAs to encoded proteins is a new breakthrough for the functional study of circRNAs. Using bioinformatics methods combined with RNA pull-down assays, we found that circRNA hsa_circ_0074834 binds to miR-942-5p and regulates its function. Studies have reported that miR-942-5p can regulate the progression of ovarian cancer^27^ and can regulate the metastasis of colon cancer^28^, but there is no research report on the role of BMSCs in the regulation of osteogenic differentiation. Our first study found that miR-942-5p can regulate the osteogenic differentiation of BMSCs. Using bioinformatics software such as Targetscan, we found that miR-942-5p can target the regulation of ZEB1 and VEGF expression. ZEB1 encodes a zinc finger transcription factor. The encoded protein likely plays a role in the transcriptional repression of interleukin 2. Mutations in this gene have been associated with posterior polymorphous corneal dystrophy-3 and late-onset Fuchs endothelial corneal dystrophy. Alternatively, spliced transcript variants encoding different isoforms have been described. We first discovered that ZEB1 regulates the osteogenic differentiation of BMSCs. There are two common methods for constructing nonunion models: (1) formation of bone defect method—a distance of the femur stump of the experimental animal of more than 6 mm is the critical value for nonunion formation. (2) Broken end activity method—an impact affects the biological stability of bone generation. (3) Tissue isolation method—the spacer is placed on the fracture end, the bone crawling process is hindered, the bone repair potential is weakened, and the formed bone is noncontinuous. (4) Excisions of the periosteum and the scraping bone marrow method—the soft tissue and periosteum are destroyed to cause blood supply damage, weakening the healing ability of bone tissue. (5) The drug induction method—mainly involves the application of drugs that inhibit angiogenesis or nonsteroidal anti-inflammatory drugs, all of which inhibit the formation of blood cells to inhibit cell proliferation. The above methods lead to nonunion by destroying bone repair or angiogenesis. During bone remodeling, BMSCs undergo osteogenesis and are vascularized. We found that hsa_circ_0074834 regulates VEGF expression during osteogenic differentiation of BMSCs. Vascular coupling. Hsa_circ_0074834 regulates the expression of VEGF by a ceRNA mechanism. This study found that hsa_circ_0074834 may be a new target for the treatment of nonunion.

## Conclusion

In this study we found that hsa_circ_0074834 is down regulated in hBMSCs isolated from bone nonunion patients, which promote ZEB1 and VEGF expression by down regulate the function of miR-942-5p. Our results explained, at least in part, the reduced expression of hsa_circ_0074834 is an important cause of nonunion, and suggested a new therapeutic strategy for the treatment of bone nonunion.

## Supplementary information

supplementary figure legends

Supplementary Figure Legends Clean

Figure S1

Figure S2

Figure S3

Figure S4
