# Supplementary material for: CircRNA hsa_circ_0074834 promotes the osteogenesis-angiogenesis coupling process in bone mesenchymal stem cells (BMSCs) by acting as a ceRNA for miR-942-5p
Source: Cell Death Dis. 2019 Dec 5;10(12):932. doi: 10.1038/s41419-019-2161-5 (PMC6895238; doi:10.1038/s41419-019-2161-5)
Supplement: Supplementary file 1 — supplementary figure legends [file 41419_2019_2161_MOESM1_ESM.docx]

**Supplementary Figure Legends**

Figure S1 Identification of hBMSCs. The expression of hBMSCs surface marker of positive(CD90, CD105, CD44 and CD29) and negative(CD34 and CD45) were analyzed by FACS.

Figure S2 The effect of TTC1 on the osteogenesis of hBMSCs. (A) ALP staining after BMSCs were transfected with lentiviruses pLVX-Vector, pLVX-NM_001282500.1 and pLVX-NM_003314.3 on the 7^th^ day and 14^th^ day. (B) ALP and Alizarin red staining after BMSCs were transfected with lentiviruses pLVX-Vector, pLVX-NM_001282500.1 and pLVX-NM_003314.3 on the 14^th^ day and 21^th^ day. Quantitative analysis of (C) ALP and (D) Alizarin red staining after BMSCs were transfected with pLVX-Vector, pLVX-NM_001282500.1 and pLVX-NM_003314.3. (J) Western blot analysis of TTC1 protein expression during osteogenesis of hBMSCs on the 7^th^ day and 14^th^ day.

Figure S3 CCK-8 assay detect the effect of hsa_circ_0074834 on the proliferation of hBMSCs.

Figure S4 miR-942-5p binding to the 3’-UTR of ZEB1 and VEGF mRNA. (A) The predicted binding site of miR-942 at hsa_circ_0074834. (B) The predicted binding site of miR-942 at 3’-UTR of ZEB1 and VEGF mRNA and the mutation sequence of predicted binding site of miR-942 at 3’-UTR of ZEB1 and VEGF mRNA. (C) Luciferase activity of psiCHECK2-VEGF-3’-UTR-wt and psiCHECK2-VEGF-3’-UTR-mut upon cotransfection with miRNA mimics NC or miR-942-5p mimics in 293T cells. (D) Luciferase activity of psiCHECK2-ZEB1-3’-UTR-wt and psiCHECK2- ZEB1-3’-UTR-mut upon cotransfection with miRNA mimics NC or miR-942-5p mimics in 293T cells. The results are presented as the mean ± SD. *p < 0.05, **p < 0.01.
