# Supplementary figures and images for: CircRNA hsa_circ_0074834 promotes the osteogenesis-angiogenesis coupling process in bone mesenchymal stem cells (BMSCs) by acting as a ceRNA for miR-942-5p
Source: Cell Death Dis. 2019 Dec 5;10(12):932. doi: 10.1038/s41419-019-2161-5 (PMC6895238; doi:10.1038/s41419-019-2161-5)

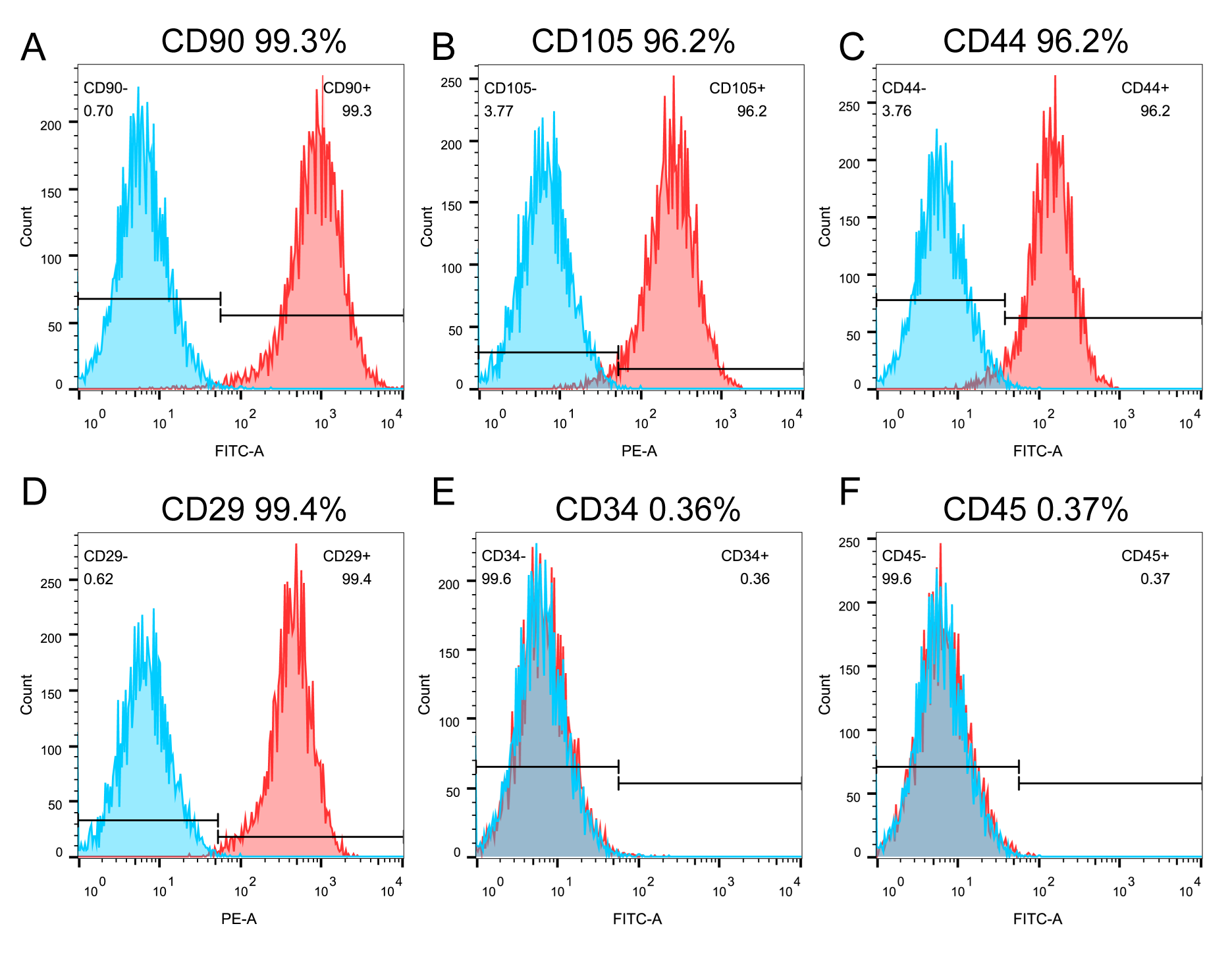

Supplement: Supplementary file 3 — Figure S1 [file 41419_2019_2161_MOESM3_ESM.tif]

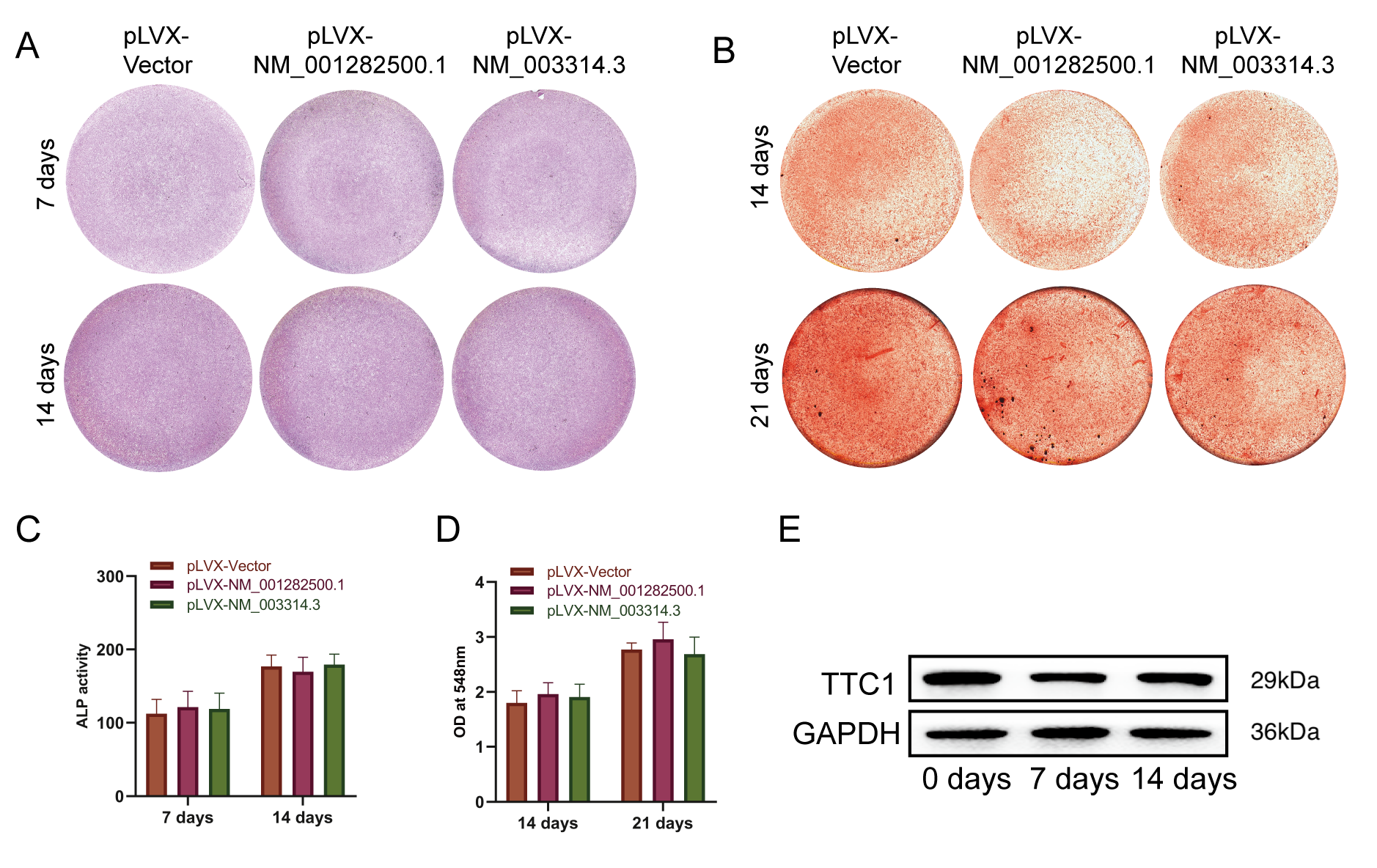

Supplement: Supplementary file 4 — Figure S2 [file 41419_2019_2161_MOESM4_ESM.tif]

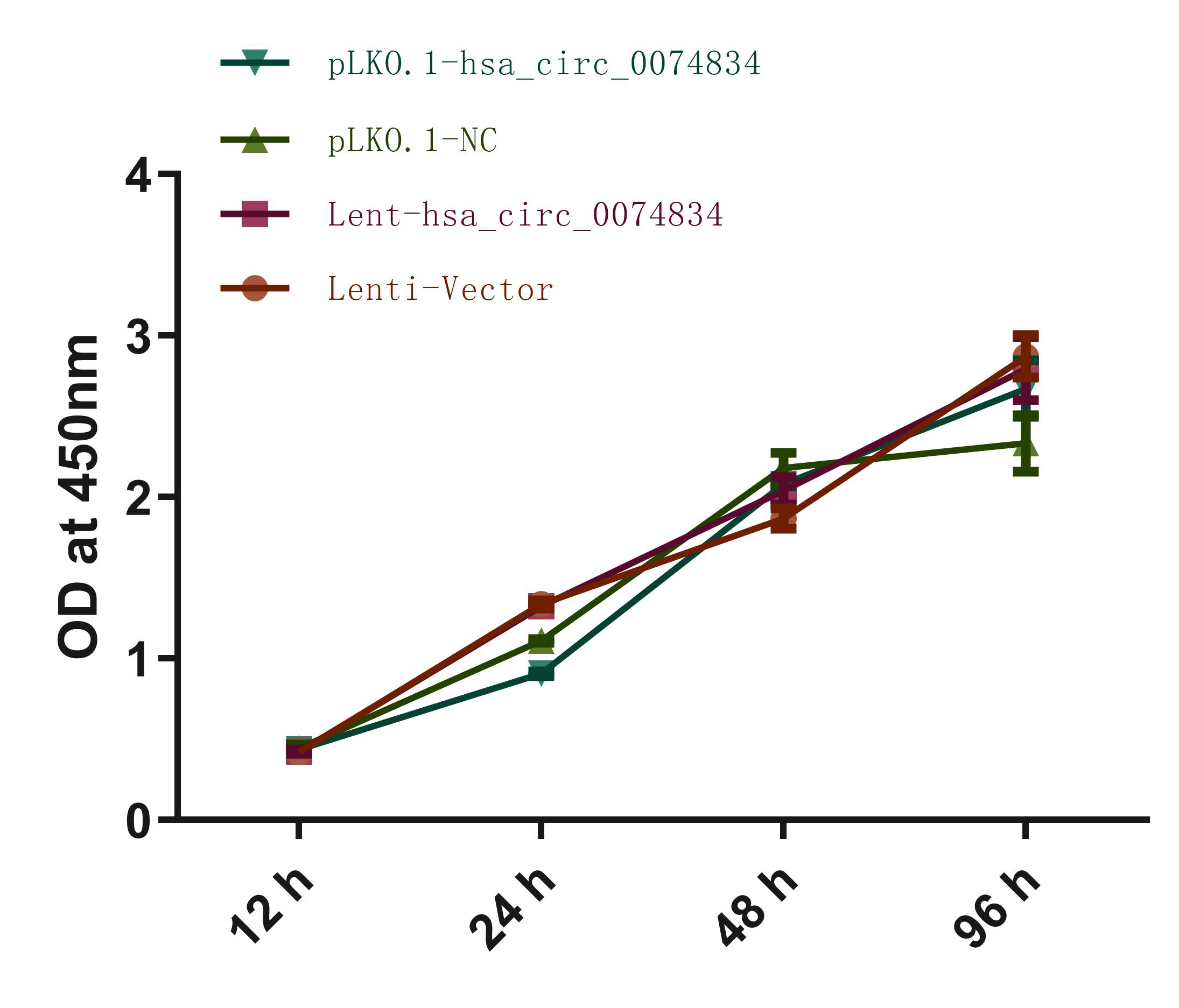

Supplement: Supplementary file 5 — Figure S3 [file 41419_2019_2161_MOESM5_ESM.tif]

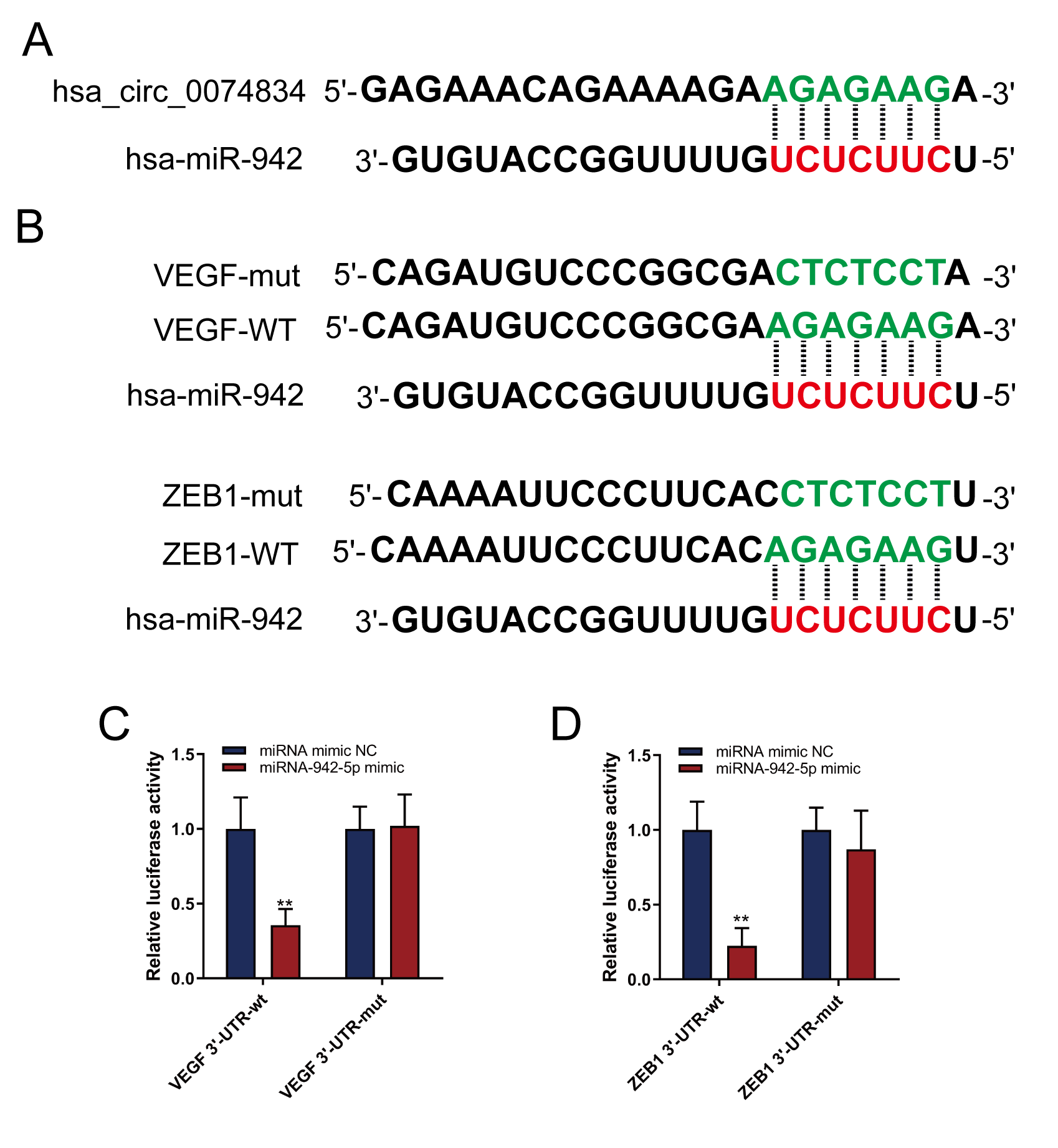

Supplement: Supplementary file 6 — Figure S4 [file 41419_2019_2161_MOESM6_ESM.tif]
